# Engaging in Advocacy to Promote Policy Change: Incorporating Advocacy-Informed Research and Applying the PRISM Implementation Science Model

**DOI:** 10.1007/s11606-026-10169-0

**Published:** 2026-01-23

**Authors:** Lilia Cervantes, Katherine Rizzolo, Apoorva Ram, Russell E. Glasgow, Mark Earnest

**Affiliations:** 1 Department of Medicine, University of Colorado, Aurora, CO USA; 2 Section of Nephrology, Boston Medical Center, Boston, MA USA; 3 Evans Center for Implementation and Improvement Sciences, Boston University, Boston, MA USA; 4 Department of Family Medicine and ACCORDS Research Center, University of Colorado Anschutz Medical Campus, Aurora, CO USA

## INTRODUCTION

The drive to engage in research often extends beyond academic curiosity to encompass a critical pursuit—advocacy to catalyze meaningful policy change^1–3^. Advocacy-informed research (AIR) is research designed and conducted to inform or influence the policies and paradigms that maintain or improve individual and population health outcomes^4^. In terms of theory, frameworks, or training, there is little available on how to conduct AIR for maximal impact. A comprehensive framework that can assess, evaluate, and implement advocacy activities, including AIR, in partnership with the community, would promote rigorous research and advocacy in parallel^5,6^.

Implementation science, also known as dissemination and implementation research, focuses on contextual and systematic strategies for designing, executing, and evaluating the effectiveness and implementation of evidence-based interventions to change practice^7^. Implementation science thus sits at the intersection of research and policy change; however, few implementation science frameworks offer specific guidance into the “where, how, and when” of advocacy activities^8^. Utilizing implementation science theories, frameworks, and models facilitates an understanding of the multi-level and multi-sector stakeholder dynamics and settings that underpin advocacy—ensuring that AIR findings translate into actionable, equitable, and sustainable advocacy activities to catalyze policy change^9–12^.

In prior work, we developed a model that breaks down the complex process of advocacy into distinct components—levels, settings, and advocacy strategies—serving as both a practical guide for planning advocacy efforts and equipping clinicians with advocacy strategies to drive policy change^13^. Recognizing that AIR promotes effective advocacy, this manuscript aims to expand our original advocacy model to include AIR. Second, we apply the Practical Robust Implementation and Sustainability Model (PRISM), a widely used implementation science model, to implement the advocacy model. PRISM was selected for its emphasis on equity, multi-level and multi-sector community perspectives, and the external environment (e.g., public opinion, awareness), making it well-suited for planning, implementing, and evaluating advocacy in diverse, real-world settings^14,15^. The objectives of this manuscript are to (1) summarize the expanded advocacy model to include AIR^13^; (2) apply the PRISM to implement the advocacy model; and (3) provide a practical example to illustrate this application.

## EXPANDED ADVOCACY MODEL

**Figure 1:**
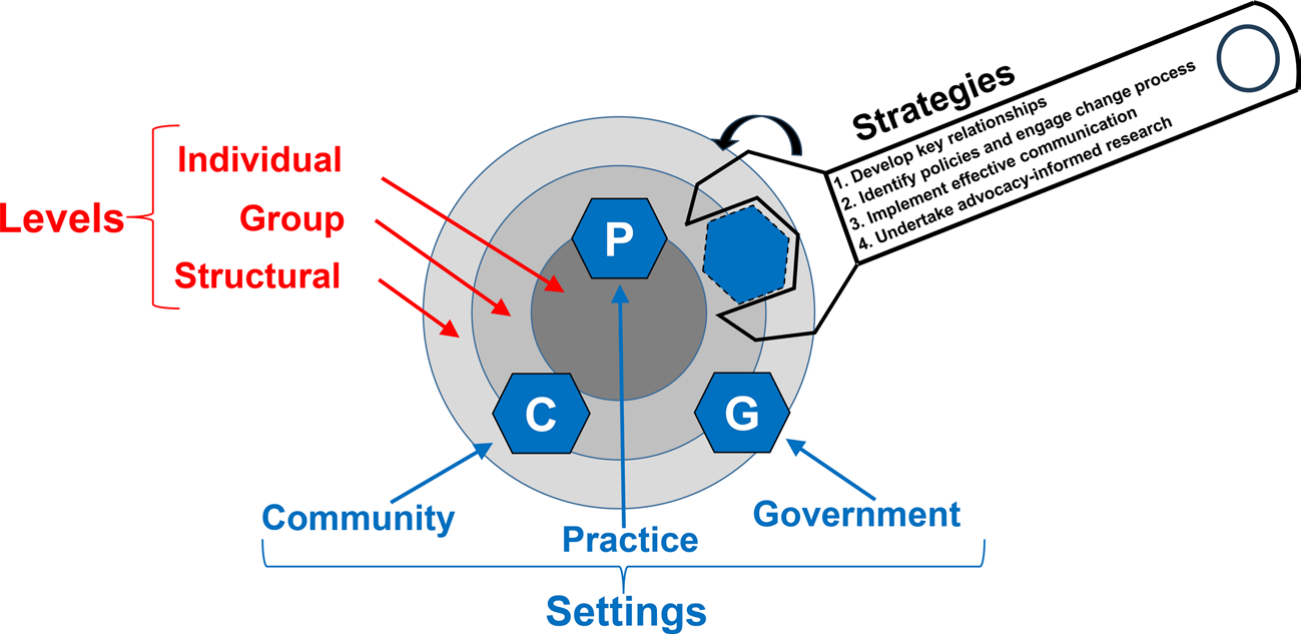
Advocacy model.

Our previously published advocacy model describes advocacy using three components: levels, settings, and strategies (termed *skills* in the original model). The advocacy model (Fig. 1) illustrates how advocacy strategies—represented within the wrench—apply pressure to influence change across clinical practice, community, and governmental settings (represented as lug nuts) to promote patient health and well-being at multiple levels: individual, group, or structural (i.e., a population at the structural level). Unlike other models that emphasize spheres of influence, this advocacy model integrates domains (i.e., settings) of influence with advocacy strategies and levels of change, creating a multidimensional, actionable framework that enables tailored advocacy across many contexts^16^. In this manuscript, we add more detail about the advocacy strategies and expand the model by adding one advocacy strategy—AIR.

### Advocacy Levels

Our model describes three levels of advocacy—individual, group (modified from *adjacent* in the model), and structural (i.e. a population)—which are represented as concentric circles. At the individual level, for example, one may work to fix a challenge faced by a single individual (e.g., provide housing resources for an individual with complex health needs who is at risk of losing their home). Group advocacy generates a solution for a group of people (e.g., ensure high-quality language interpretation for people who report limited English proficiency at a dialysis center). Structural advocacy eliminates a challenge for a population (e.g., modifying Medicaid policy to ensure that uninsured individuals in the state receive healthcare coverage for kidney transplantation). At every level, the goal remains to advance or maintain the well-being of patients—whether for a single patient, a group of patients, or at the structural level for an entire population. Many structural changes begin with efforts at the individual and group levels progressing to the population level as advocates gain an understanding of the issues and build key relationships needed to expand their efforts.

### Advocacy Setting

There are three settings where advocacy may occur—clinical practice, community, and governmental. The settings are represented as “lug nuts” to emphasize settings in which influence or pressure is necessary to create change. The practice setting includes clinical or healthcare settings. For example, one may work to ensure there are sufficient social workers in a hospital to effectively identify and address patient social needs prior to hospital discharge. The community setting includes non-governmental, community-based organizations and other public or private institutions. In a community setting, for example, one may develop partnerships with local food banks to address food insecurity for people with kidney failure. The governmental setting includes municipal, state, or federal policy. Advocacy in this setting may include interactions with governmental representatives, decision-makers, and legislators to change local, state, or federal policy. Individual- and group-level change frequently occurs in a single setting (e.g., practice or community) while structural-level change most often requires coordinated efforts across multiple settings.

### Advocacy Strategies

There are four advocacy strategies. Three of them are from the original advocacy model, and we have added a fourth. The three original advocacy strategies include (1) develop key relationships; (2) identify policies and engage change processes; and (3) implement effective communication. They are represented in a wrench to emphasize how they generate influence or force in the various settings to effect change. First, developing key relationships requires connecting with individuals and communities affected by the advocacy issue, in addition to allies, opponents, and people with power and influence^17,18^. Second, to advocate effectively, one must identify both the existing policies and the processes through which these policies can be changed. Lastly, building effective communication requires careful consideration of the audience, the structure of the message, and the communication modality^19^. As one meets with these key groups, it becomes clear which data gaps require additional research.

For this reason, we have added advocacy-informed research (AIR) to the model. AIR adheres to the same standards of ethical and methodological rigor as any research. Thus, AIR does not mean advocacy-biased. Scientific integrity requires pre-specification of research aims and analytic plans, attention to trustworthiness in qualitative research, and vigilance against interpretive drift toward any political ideology.  Such research might quantify the economic impacts of the status quo or of a proposed solution to a problem—as well as identify who benefits and who suffers because of a policy. AIR both informs and is informed by key relationships and policies leveraging influence over advocacy settings. For example, key relationships and an understanding of policy may inform gaps in research; the research itself may inform how a policy should be defined or identify additional necessary key relationships. The research findings are then used for advocacy through effective communication that considers the values and lived experiences of allies and those who resist.  AIR findings must also be communicated with humility and objectivity so that they earn and maintain public trust.

## PRISM

The PRISM is a broadly utilized implementation science framework focused on identifying contextual factors that influence implementation strategies and the RE-AIM (Reach, Effectiveness, Adoption, Implementation, and Maintenance) outcomes of a healthcare intervention, guideline, or policy. According to a 2022 review, PRISM has over 200 published applications across a diverse body of literature, indicating its breadth, generalizability, and accessibility of use^15^. The PRISM is comprised of two primary components: the four contextual domains and the RE-AIM outcomes. The four contextual domains include (1) multi-level and multi-sector recipient perspectives on the intervention being studied; (2) multi-level and multi-sector recipient characteristics; (3) the external environment; and (4) the implementation and sustainability infrastructure.

**Figure 2:**
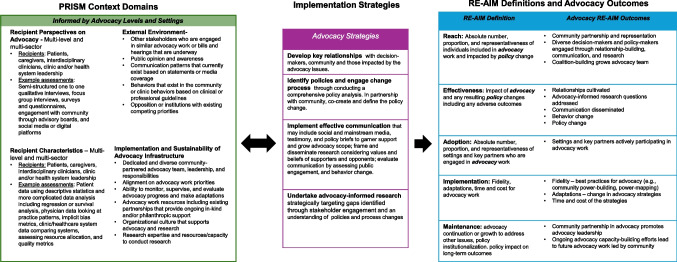
Application of PRISM for advocacy. Adapted from Glasgow et al.^13^.

Contextual assessment of multi-level and multi-sector perspectives and characteristics can enhance the effectiveness of evidence-based interventions; similarly, assessing the current context of an advocacy issue strengthens planning by informing advocacy strategies and clarifying desired advocacy outcomes. Applying PRISM to advocacy illustrates how levels and settings within the advocacy model inform its four contextual domains (Fig. 2)^15^. For example, PRISM emphasizes diverse representation; thus, incorporating multi-level and multi-sector perspectives on the advocacy issue may involve engaging patients, caregivers, clinicians, and healthcare system leaders. To further capture heterogeneity, patient engagement may intentionally include individuals across racial and ethnic backgrounds, language proficiency, immigration status, age, gender, and sex. Partnering with the community to understand their perspectives on an advocacy issue can provide insight into what's important as well as the barriers and facilitators to engagement in advocacy work. To assess recipient characteristics within PRISM for an advocacy issue, one might assess the socioeconomic factors, which influence a community’s capacity to engage in advocacy, and geographic distribution, which informs where advocacy efforts should be concentrated. Assessing the external environment within PRISM informs advocacy strategies by identifying contextual factors that shape opportunities and barriers for advocacy. This includes evaluating people engaged in similar advocacy efforts, pending legislation, public opinion and awareness, and communication patterns reflected in media coverage or organizational statements. Additional considerations include community behaviors and potential opposition or competing priorities. Understanding these dynamics enables advocacy planning that aligns strategies with existing momentum, anticipates resistance, and leverages coalition-building to maximize impact. Lastly, assessing implementation and sustainability infrastructure within PRISM informs advocacy planning by identifying existing resources, organizational capacity, and mechanisms for monitoring and evaluation of the advocacy work^20^. This includes evaluating current advocacy networks, staffing, funding streams, and technical expertise. Understanding these elements helps determine feasibility and  the design of advocacy strategies that leverage existing strengths and build capacity for long-term sustainability of advocacy work.

A pragmatic assessment of the four PRISM contextual domains informs the advocacy model’s “advocacy strategies” (replaces the more general *implementation strategies* in PRISM), which are defined as “the actions taken to enhance adoption, implementation, and sustainability” of advocacy, and RE-AIM outcomes. RE-AIM provides a structured approach to evaluating advocacy outcomes. *Reach*, for example, evaluates the number and representativeness of community members who are meaningfully engaged in the advocacy work including community partnerships, and diverse decision-makers. *Effectiveness* examines the impact of advocacy and resulting policy change, considering relationships cultivated, dissemination of communication, and behavior change. *Adoption* includes an assessment of the settings and key partners who actively participate in advocacy work. *Implementation* focuses on fidelity to best practices, adaptation to advocacy strategies, and the time and cost required for advocacy activities. *Maintenance*considers the sustainability of advocacy efforts including ongoing capacity-building, and leadership development^14,20–22^.

## EXAMPLE APPLICATION

By expanding the advocacy model to include AIR and putting the model into practice with PRISM, we can use implementation science methodologies to conduct a robust assessment of contextual domains that inform advocacy strategies and RE-AIM outcomes for advocacy. This facilitates a critical evaluation of how advocacy strategies affect the implementation, equity, and sustainment of advocacy (i.e., increased capacity and engagement promotes sustained community advocacy).

To illustrate the use of the advocacy model (Fig. 1) and the application of PRISM (Fig. 2), we provide an example by retrospectively applying the model and PRISM to a real-world advocacy effort aiming to change policy—the expansion of healthcare coverage for maintenance hemodialysis for uninsured immigrants with kidney failure^23^. In Colorado and prior to 2019, uninsured immigrants with kidney failure relied solely on emergency dialysis (i.e., dialysis only after presenting critically ill to an emergency department). The advocacy goal was to expand healthcare coverage for thrice-weekly maintenance hemodialysis, the standard of care for people with kidney failure.

We partnered with a community of patients, caregivers, and interdisciplinary clinicians to engage in advocacy and conduct AIR. Our team initially focused on advocacy for a single patient (individual level) at the safety-net hospital (clinical practice setting)^3^. Through contextual assessment, the scope evolved to the governmental setting, aiming to expand access statewide at the structural level for the population. As such, the setting of advocacy also shifted from the safety-net hospital setting (practice setting) to the state Medicaid agency (governmental setting), requiring engagement with policymakers. In applying PRISM, the process would begin with assessing contextual, multi-level, and multi-sector perspectives on the advocacy issue. To achieve this, we established a community steering committee (CSC) composed of patients, caregivers, clinicians, and representatives from community-based organizations. In addition to the CSC’s input, we conducted qualitative interviews with uninsured immigrants who relied on emergency dialysis, their caregivers, and clinicians. We found that patients face significant physical and psychosocial distress, their caregivers face emotional exhaustion, and clinicians experience several drivers of burnout^24–26^. Informal engagement with hospital leadership revealed interest in participating in advocacy efforts. In assessing characteristics, we found that there were an estimated 80–100 uninsured immigrants in Colorado with kidney failure and the majority received care at the safety-net hospital. Treatment occurred through emergency dialysis, requiring substantial resources including weekly emergency department (ED) visits, inpatient stays, multidisciplinary physician evaluations, and diagnostic testing such as laboratory draws and imaging. External environment assessment revealed that, at the safety-net hospital (practice setting), high costs were driven by emergency department (ED) visits and hospitalizations which also strained the hospital by limiting ED capacity. At the governmental setting, the Joint Budget Commission was actively exploring strategies to reduce state healthcare expenditures and, composed primarily of Democratic legislators, demonstrated a favorable disposition toward expanding coverage. With respect to our implementation and sustainability infrastructure, our CSC and a growing and diverse coalition of patients, clinicians, and community members formed an advocacy team that was strongly aligned with expanding access to standard dialysis.

The robust contextual assessment informed the advocacy strategies. First, we developed key relationships with the state Medicaid agency and JBC. Second, we identified policies and engaged in change processes; this was critical not only for investigating and defining policy consequences, but also for identifying policy alternatives and their potential costs and benefits^27^. The team gained an understanding of existing federal policy including the Emergency Medical Treatment and Active Labor Act (EMTALA). Because the federal government defers to states to define which medical conditions and services qualify under Emergency Medicaid, several states had already expanded their programs to include dialysis—creating a precedent that informed our advocacy. Third, we implemented effective communication tailored to the needs and values of diverse audiences. Lastly, key relationships and an understanding of the policies informed research gaps including the need for an assessment of quality of life, mortality, and an economic analysis. Our team undertook AIR and documented a 14-fold higher mortality, lower quality of life, and higher symptom burden among uninsured immigrants who rely on emergency versus standard dialysis. An economic analysis demonstrated over 15 million dollars would be saved annually with the proposed healthcare policy change^28,29^.

Applying the RE-AIM to advocacy outcomes: For *Reach*, our partners, including patients, clinicians, decision-makers, and others in the community partnered on advocacy efforts.   Our community-led efforts included research, dissemination of findings through media, and coalition-building. For *Effectiveness*, we achieved expansion of Emergency Medicaid benefits for regular hemodialysis^23^. Regarding *Adoption,* two healthcare systems, non-profit community-based organizations, and other key partners joined us and supported the advocacy work. For *Implementation*, we established a community steering committee (CSC) that met regularly, fostered partnerships, and promoted community power-building. Finally, for *Maintenance,*sustained advocacy by the CSC and an expanding coalition of clinicians, decision-makers, and policymakers drove additional policy changes including expanded benefits for home dialysis and outpatient COVID-19 care^23,30^. A decade later, the CSC continues to meet regularly and partners on advocacy and AIR.

## DISCUSSION

Research that illuminates a path toward better health yet catalyzes no change represents wasted resources and lost opportunity that can often be measured in lives and suffering. Advocacy and implementation science represent two closely allied concepts and strategies that can maximize the impact of research and minimize the risk of lost opportunities to improve human health. In this paper, we expand our prior advocacy model (Fig. 1) to incorporate AIR and illustrate how advocacy strategies (represented within the wrench) apply pressure to influence change across clinical practice, community, and governmental settings (represented as lug nuts) to promote patient health and well-being at multiple levels. We also apply the PRISM (Fig. 2), an established implementation science model, to implement the advocacy model, offering a comprehensive contextual assessment that informs planning, implementation, and evaluation of advocacy work to promote policy change.

AIR combines rigorous, reproducible science with advocacy to influence policies that improve the health and well-being of individuals and populations. AIR rejects the false dichotomy between research and advocacy, transforming research from isolated knowledge into action and ensuring that evidence-based findings are translated into meaningful policy change. To design AIR, it is essential to conduct a contextual assessment that considers multi-level and multi-sector perspectives and characteristics, as well as the external political environment. By understanding the nuanced, political, and organizational environments, we can better formulate AIR questions that are relevant and actionable within real-world settings.

Implementation science provides a valuable framework to systematically assess contextual factors, enabling us to identify how, where, and when advocacy efforts can be most effective^31^. Implementation science methods can thus strengthen advocacy work, including AIR, by ensuring that contextual nuances are central components of advocacy efforts, ultimately improving the relevance, scalability, sustainability, and impact of advocacy. However, few implementation science frameworks explicitly outline how to implement advocacy across different contexts to facilitate advocacy and ultimately policy change. While implementation science frameworks such as Consolidated Framework for Implementation Research (CFIR) and the Exploration Planning, Implementation, and Sustainment (EPIS) models have been utilized or applied to inform advocacy at the structural level for specific contexts, to our knowledge, this manuscript illustrates the first adaptation of a well-known and broadly utilized implementation science framework to inform advocacy work^32–35^. We used PRISM in this article due to its accessibility and breadth, and our experience with PRISM^15^.

In the example provided, advocacy was aimed at addressing the lack of healthcare benefits for maintenance hemodialysis for uninsured immigrants. A key reflection from our work is that qualitative findings—through patient storytelling—often resonated more strongly with policymakers, media, and initial opponents of change than quantitative data alone. Narrative storytelling through qualitative research is a critical research method for advocacy because it elevates the voices of those most affected, providing a human-centered perspective often absent in quantitative data. By capturing lived experiences, qualitative studies give patients, caregivers, and others a platform in academic literature and create compelling narratives that can be leveraged in policy briefs, testimonies, and media. These narratives are powerful for influencing behavior change among decision-makers and policymakers, as they contextualize real-world consequences of policies that might otherwise be reduced to financial or operational metrics. Our team’s experience shows that such narratives not only attract attention from policymakers and media but also influence people who are initially resistant to policy reform.

Our team engaged in identifying the relevant policies and change processes, which informed the development of a policy brief synthesizing evidence on the impact of emergency dialysis on patient outcomes. This brief delineated the specific Emergency Medicaid policy changes required and provided actionable recommendations to policymakers and decision-makers. After clarifying the policy pathways, we focused on understanding the relationships that influence decision-making. To achieve this, one can employ power mapping—a visual political and social science tool designed to reveal underlying power structures. Power mapping organizes key actors, including decision-makers, affected communities, and involved organizations, along two dimensions: their level of influence and their alignment with the proposed policy change. This approach enables advocates to identify strategic relationships, understand how power is distributed, and leverage these dynamics to advance policy reform^17^. Our team’s advocacy efforts grew our coalition, resulting in several policy changes that expanded healthcare benefits. With respect to RE-AIM, our advocacy efforts achieved broad dissemination through peer-reviewed publications, webinars, conference presentations, radio, and other media, extending reach to clinicians, policymakers, and community members. This visibility facilitated policy adoption in multiple states, expanding dialysis access for uninsured immigrants with kidney failure^30^. Following policy change, PRISM can be applied again or used iteratively^36^. One can utilize PRISM after changing policy in order to evaluate the contextual determinants of policy implementation and evaluation of reach and maintenance (sustainment) of the policy. In the example, an understanding of the policy’s context motivated additional research and coalition-building to demonstrate that the policy change was fiscally sustainable and supported by clinicians and that its implementation could be further refined and improved^29,37^.

Clinician engagement in health policy advocacy is an essential component of medical professionalism. Beyond delivering clinical care, clinicians have an ethical obligation to address health inequities and unjust laws that harm patients. Advocacy represents an extension of a physician’s duty to act in the best interest of patients, particularly when systematic barriers perpetuate preventable suffering. The American Medical Association’s Principles of Medical Ethics underscores this dual responsibility: clinicians must respect the law while advocating for policy changes that advance patient welfare^38^. Despite this mandate, clinicians and clinician researchers lack training in advocacy or in conducting AIR to catalyze policy change^13^^,39^. Our expanded advocacy model and application of PRISM provide a structured approach for implementing advocacy within a robust, accessible, implementation science model. By leveraging implementation science principles, physicians and researchers can partner with the community to design, implement, and evaluate advocacy work^25,40,41^.

Our framework has limitations. First, to our knowledge, PRISM has not been proactively applied to guide advocacy. Future research should prioritize pilot testing the application of the PRISM for advocacy to assess its effectiveness in facilitating policy change. This pilot testing can identify specific challenges while also uncovering opportunities for improvement. By engaging researchers motivated to drive policy changes through advocacy, iterative adjustments to the PRISM framework can be made based on pilot feedback, enhancing its applicability^42^. These iterative changes will not only refine the application of PRISM but also foster a collaborative environment where researchers can share insights and strategies, ultimately leading to more impactful adaptations that advance health equity. Of note, promoting this type of science is becoming increasingly important as funding agencies such as the National Institutes of Health publish requests for applications that explicitly state that research must be conducted with the intention to improve systems and structures^43,44^. Lastly, our framework is focused on the end goal of conducting implementation science-guided advocacy to promote policy change; we acknowledge that this framework may be utilized for a broad range of advocacy activities, such as the mobilization of public voices, increasing public and political awareness and support, improving collaborative action among partners, among others^45^.

## CONCLUSION

We define AIR and expand our advocacy model to include AIR as a core advocacy strategy for advancing advocacy. The model uses the metaphor of a wrench applying pressure to lug nuts—clinical practice, community, and governmental settings—to drive change. We also apply PRISM to guide planning, implementation, and evaluation of advocacy efforts. Incorporating advocacy into an existing implementation science framework strengthens the bridge between evidence, advocacy, and policy change by embedding equity-driven priorities, amplifying community voice, and enhancing the likelihood that research will lead to meaningful and sustained advocacy work and policy change.

## Data Availability

This study does not utilize any primary data. Therefore, there is no data availability relevant to this work.
